# Supplementary material for: Evaluation of clinical utility in emulated clinical trials
Source: Eur J Epidemiol. 2026 May 4;41(7):829–39. doi: 10.1007/s10654-026-01385-3 (PMC13572323; doi:10.1007/s10654-026-01385-3)
Supplement: Supplementary file 1 [file 10654_2026_1385_MOESM1_ESM.pdf]

Supplementary Material for "Evaluation of clinical utility in emulated clinical trials"

Johannes Hruza, Arvid Sjölander, Erin Gabriel, Samir Bhatt, Michael Sachs

December 9, 2025

1 Identifiability

If a distribution on which an intervention has been performed can be reduced to an observational distribution, then the distribution is called identifiable. In our setting we have this favourable property. Let $P(y; \sigma_T)$ be the distribution of Y where we have intervened on T by the conditional intervention $\sigma_T = do(T = f(\boldsymbol{Z}))$. We start by factorising the distribution:

$$P(y; \sigma_T) = \sum_{t,\boldsymbol{z}} P(y \mid t, \boldsymbol{z}, \sigma_T) P(t \mid \boldsymbol{z}; \sigma_T) P(\boldsymbol{z}, \sigma_T) \tag{1}$$

$$= \sum_{t,\boldsymbol{z}} P(y \mid t, \boldsymbol{z}) P(t \mid \boldsymbol{z}; \sigma_T) P(\boldsymbol{z}). \tag{2}$$

By rule 2 of [Correa and Bareinboim(2020)], we can rewrite $P(y \mid t, \boldsymbol{z}, \sigma_T) = P(y \mid t, \boldsymbol{z})$ if we consider $\sigma'_T = \emptyset$ and observe that $(Y \perp\!\!\!\perp T \mid \boldsymbol{Z})$ in $G_{\underline{T}}$ and $G_{\sigma_T \underline{T}}$. Rule 3 (with $\sigma'_T = \emptyset$) justifies $P(\boldsymbol{z}, \sigma_T) = P(\boldsymbol{z})$ by the independence of $(\boldsymbol{Z} \perp\!\!\!\perp T)$ in $G_{\overline{T}}$ and $G_{\sigma_T \overline{T}}$.

Let $\mathbb{1}(\cdot)$ be the indicator function that is one if the condition in the brackets is satisfied and otherwise zero. In the framework of sigma calculus the decision rule f is a conditional intervention hence $P(t \mid \boldsymbol{z}; \sigma_T) = \mathbb{1}(t = f(\boldsymbol{z}))$ which implies identifiablility as we have:

$$P(y; \sigma_T) = \sum_{t,\boldsymbol{z}} P(y \mid t, \boldsymbol{z}) \mathbb{1}(t = f(\boldsymbol{z})) P(\boldsymbol{z}) \tag{3}$$

2 Deriving IPW estimator

There are several ways of estimating the expected outcome under the decisions rule, which can be seen via identifiabilty property equations of [Correa and Bareinboim(2020)], as we demonstrate below.

First, we derive an inverse probability of treatment weighted estimator following the identifiabilty property equations derived above. We observe that

$$E\left[Y \frac{\mathbb{1}(f(\boldsymbol{Z}) = T)}{P(T = f(\boldsymbol{Z}) \mid \boldsymbol{Z})}\right] = E[Y, \sigma_T].$$

This can be seen via identification property (3), and assume without loss of

generality that all variables are discrete, since:

$$
\begin{aligned}
E[Y, \sigma_T] &= \sum_y y P(y; \sigma_T) && (4) \\
&= \sum_y \sum_{t, \boldsymbol{z}} y P(Y = y \mid T = t, \boldsymbol{Z} = \boldsymbol{z}) \mathbb{1}(t = f(\boldsymbol{z})) P(\boldsymbol{Z} = \boldsymbol{z}) && (5) \\
&= \sum_{y,t,\boldsymbol{z}} y P(Y = y \mid T = t, \boldsymbol{Z} = \boldsymbol{z}) \mathbb{1}(t = f(\boldsymbol{z})) P(\boldsymbol{Z} = \boldsymbol{z}) \frac{P(T = t \mid \boldsymbol{Z} = \boldsymbol{z})}{P(T = t \mid \boldsymbol{Z} = \boldsymbol{z})} && (6) \\
&= \sum_{y,t,\boldsymbol{z}} y P(y, t, \boldsymbol{z}) \mathbb{1}(t = f(\boldsymbol{z})) \frac{1}{P(T = t \mid \boldsymbol{Z} = \boldsymbol{z})} && (7) \\
&= \sum_{y,t,\boldsymbol{z}} y \frac{\mathbb{1}(t = f(\boldsymbol{z}))}{P(T = t \mid \boldsymbol{Z} = \boldsymbol{z})} P(y, t, \boldsymbol{z}) && (8) \\
&= E_{Y,T,\boldsymbol{Z}} \left[Y \frac{\mathbb{1}(f(\boldsymbol{Z}) = T)]}{P(T = f(\boldsymbol{Z}) \mid \boldsymbol{Z})} \right]. && (9)
\end{aligned}
$$

If variables $\{Y, \boldsymbol{Z}\}$ are continuous, we can replace sums with integrals in the above. Regardless, T remains discrete. This directly leads to the IPW estimator:

$$\hat{E}[Y, \sigma_T] = \frac{1}{n} \sum_{\substack{i=1 \\ f(\boldsymbol{z}_i) = t_i}}^{n} \frac{y_i}{\hat{p}(T = t_i \mid \boldsymbol{z}_i)} \tag{10}$$

The derivation of 4 gives rise to a suggested G-computation estimator, as follows

$$\hat{E}[Y, \sigma_T] = \hat{E}\{\hat{E}[Y | T = f(\boldsymbol{z}_i), \boldsymbol{Z}]\} = \frac{1}{n} \sum \hat{Y}_i(T = f(\boldsymbol{z}_i)), \tag{11}$$

where $\hat{Y}_i(T = f(\boldsymbol{z}_i)) = g(T = f(\boldsymbol{z}_i), \boldsymbol{z}_i; \hat{\gamma})$ for some known model with known link function g, where it is assumed that $E(Y | t, \boldsymbol{z}) = g(t, \boldsymbol{z}; \boldsymbol{\gamma})$.

More standard derivations of IPW and G-computation estimators are possible by considering $\mathbb{1}(T = f(\boldsymbol{z}))$ as the binary exposure variable. The standard IPW and G-computation estimators are as follows:

$$\hat{E}[Y, \sigma_T] = \frac{1}{n} \sum_{\substack{i=1 \\ f(\boldsymbol{z}_i) = t_i}}^{n} \frac{y_i}{\hat{p}(\mathbb{1}(t_i = f(\boldsymbol{z}_i)) = 1 \mid \boldsymbol{z}_i)}, \tag{12}$$

for the IPW estimator, and

$$\hat{E}[Y, \sigma_T] = \hat{E}\{\hat{E}[Y | T = f(\boldsymbol{Z}), \boldsymbol{Z}]\} = \frac{1}{n} \sum \hat{Y}_i(\mathbb{1}(T = f(\boldsymbol{z}_i)) = 1), \tag{13}$$

for the G-computation estimator. Similar to before, $\hat{Y}_i(\mathbb{1}(T = f(\boldsymbol{z}_i)) = 1) = g^*(\mathbb{1}(T = f(\boldsymbol{z}_i)) = 1, \boldsymbol{z}_i, \hat{\boldsymbol{\gamma}^*})$ for some known model with known link function g^* where it is assumed that $E(Y | \mathbb{1}(T = f(\boldsymbol{z}_i)), \boldsymbol{Z}) = g^*(\mathbb{1}(T = f(\boldsymbol{z}_i)), \boldsymbol{z}; \boldsymbol{\gamma}^*)$.

Proof of the consistency follows directly from basic IPW and G-computation derivation, given that $f(Z)$ is deterministic under the standard assumptions of consistency, exchangeability (no unmeasured confounders between T and Y), and positivity. It is of note that for the IPW estimator in 12 positivity is required with regard to $\mathbb{1}(T = f(\boldsymbol{z}))$, i.e. $0 < P(\mathbb{1}(T = f(\boldsymbol{z})) = 1 \mid \boldsymbol{Z})$, this is also the positivity required for the G-computation estimator in 13. Although this positivity is theoretical, this all means practically that $\sum \mathbb{1}(t_i = f(\boldsymbol{z}_i)) > 0$ in order to use these estimators.

In contrast, the IPW estimator in 10 requires both positivitity for all levels of exposure t, i.e. $0 < P(T = t \mid \boldsymbol{Z})$, but again requires a positive number of observed subjects taking the treatment as would be assigned via the decision rule, i.e. $\sum \mathbb{1}(t_i = f(\boldsymbol{z}_i)) > 0$, or it will always take on the value zero. Finally, the G-computation estimator in 11 requires only positivity for all levels of the exposure t, and does not require a positive number of observed subjects taking the treatment as would be assigned via the decision rule. Thus, only this estimator is consistent when no subjects are observed to follow the decision rule under investigation.

2.1 Variance estimation

The variance of the four proposed estimators can be estimated as they are all M-estimators. The calculations are analogous hence we limit ourselves to one case. The implementation for the variance estimation for each estimator can be found in the supplementary R code.

Because of equation (2) from the main text and by assuming, that the propensity score model is assumed to follow a parametric model of the form $\pi(\boldsymbol{z}; \beta)$, we note that this satisfies the definition of an M-estimator. We will use the sandwich estimator for M-estimators to estimate the variance, see [Stefanski and Boos(2002)] for a detailed introduction. For the ipw_b estimator the propensity score $P(\mathbb{1}(T = f(\boldsymbol{Z})) = 1 \mid \boldsymbol{Z}) = \pi(\boldsymbol{Z})$ is modeled by $\text{logit}\{\pi(\boldsymbol{z}; \beta)\} = \beta^T \boldsymbol{z}$ and hence

$$\pi(\boldsymbol{z}, \beta) = \frac{\exp(\beta^T \boldsymbol{z})}{1 + \exp(\beta^T \boldsymbol{z})}.$$

Its likelihood given by

$$\prod_{i=1}^{n} \pi(\boldsymbol{z}_i, \beta)^{\mathbb{1}(t_i = f(\boldsymbol{z}_i))} [1 - \pi(\boldsymbol{z}_i, \beta)]^{1 - \mathbb{1}(t_i = f(\boldsymbol{z}_i))} = \prod_{i=1}^{n} \left[\frac{\exp(\beta^T \boldsymbol{z}_i) \mathbb{1}(t_i = f(\boldsymbol{z}_i))}{1 + \exp(\beta^T \boldsymbol{z}_i)} \right]$$

which has to be maximized. Taking the derivative $\frac{\partial}{\partial \beta}$ of the log likelihood gives us the first part of our stacked M-estimator equations:

$$\sum_{i=1}^{n} \begin{pmatrix} z_i^1 \\ \vdots \\ z_i^d \end{pmatrix} (\mathbb{1}(t_i = f(\boldsymbol{z}_i)) - \pi(\boldsymbol{z}_i, \beta)) = 0$$

Let $\delta_0 = E[Y(f(\boldsymbol{Z}))] - E[Y(g(\boldsymbol{Z}, \boldsymbol{U}))]$ be the true clinical utility between two counterfactual outcomes under f and g. Let π_f denote the propensity score under treatment regime f, and let the model for this be indexed with parameter β_f. Let π_g denote the propensity score under treatment regime g, and let this model be indexed with parameter β_g. By defining $\theta = (\delta, \beta_f, \beta_g)$ the estimators solve for θ the following stacked estimating equations

$$\sum_{i=1}^{n} \frac{\mathbb{1}(t_i = f(\boldsymbol{z}_i))y_i}{\pi_f(\boldsymbol{z}_i, \beta_f)} - \frac{\mathbb{1}(t_i = g(\boldsymbol{z}_i))y_i}{\pi_g(\boldsymbol{z}_i, \beta_g)} - \delta = 0 \tag{14}$$

$$\sum_{i=1}^{n} \begin{pmatrix} z_i^1 \\ \vdots \\ z_i^d \end{pmatrix} (\mathbb{1}(t_i = f(\boldsymbol{z}_i)) - \pi(\boldsymbol{z}, \beta_f)) = 0 \tag{15}$$

$$\sum_{i=1}^{n} \begin{pmatrix} z_i^1 \\ \vdots \\ z_i^d \end{pmatrix} (\mathbb{1}(t_i = f(\boldsymbol{z}_i)) - \pi(\boldsymbol{z}, \beta_g)) = 0 \tag{16}$$

Hence the estimating function is given by,

$$M(\theta) = \begin{pmatrix} \frac{\mathbb{1}(t=f(\boldsymbol{z}))y}{\pi_f(\boldsymbol{z},\beta_f)} - \frac{\mathbb{1}(t=g(\boldsymbol{z}))y}{\pi_g(\boldsymbol{z},\beta_g)} - \delta & \text{(M1)} \\ \begin{pmatrix} z^1 \\ \vdots \\ z^d \end{pmatrix} (\mathbb{1}(t = f(\boldsymbol{z})) - \pi(\boldsymbol{z}, \beta_f)) & \text{(M2)} \\ \begin{pmatrix} z^1 \\ \vdots \\ z^d \end{pmatrix} (\mathbb{1}(t = g(\boldsymbol{z})) - \pi(\boldsymbol{z}, \beta_g)) & \text{(M3)} \end{pmatrix}.$$

Let $\theta_0 = (\delta_0, \beta_{f0}, \beta_{g0})$ be the true value of $\theta = (\delta, \beta_f, \beta_g)$. Then, by M estimating theory

$$\sqrt{n} \begin{pmatrix} \hat{\delta} - \delta_0 \\ \hat{\beta}_f - \beta_{f0} \\ \hat{\beta}_g - \beta_{g0} \end{pmatrix} \xrightarrow{D} \mathcal{N}(0, \Sigma).$$

where the covariance matrix Σ is given by

$$E\left[\frac{\partial M(\theta_0)}{\partial \theta^T}\right]^{-1} \times E\left[M(\theta_0)M(\theta_0)^T\right] \times E\left[\frac{\partial M(\theta_0)}{\partial \theta^T}\right]^{-T}$$

where we have abused notation by writing $\frac{\partial M(\theta_0)}{\partial \theta^T} = \frac{\partial M(\theta)}{\partial \theta^T}|_{\theta=\theta_0}$ We start esti-

mating the "bread" matrix, for this notice that:

$$E\left[\frac{\partial M(\theta_0)}{\partial \theta^T}\right] = E\begin{pmatrix} \frac{\partial M1}{\partial \delta} & \frac{\partial M1}{\partial \beta_f} & \frac{\partial M1}{\partial \beta_g} \\ \frac{\partial M2}{\partial \delta} & \frac{\partial M2}{\partial \beta_f} & \frac{\partial M2}{\partial \beta_g} \\ \frac{\partial M3}{\partial \delta} & \frac{\partial M3}{\partial \beta_f} & \frac{\partial M3}{\partial \beta_g} \end{pmatrix}$$

$$= E\begin{pmatrix} -1 & \frac{\partial M1}{\partial \beta_f} & \frac{\partial M1}{\partial \beta_g} \\ 0 & \frac{\partial M2}{\partial \beta_f} & 0 \\ 0 & 0 & \frac{\partial M3}{\partial \beta_g} \end{pmatrix}$$

where $\frac{\partial M1}{\partial \beta_f} = (z^1, \cdots, z^d)[(\mathbb{1}(t = f(\boldsymbol{z}))y)(\frac{1}{\pi(\boldsymbol{z}, \beta_f)} - 1)] \in \mathbb{R}^{1\times d}$ similarly we have that

$\frac{\partial M1}{\partial \beta_g} = (z^1, \cdots, z^d)[(\mathbb{1}(t = g(\boldsymbol{z}))y)(1 - \frac{1}{\pi(\boldsymbol{z}, \beta_{g0})})] \in \mathbb{R}^{1\times d}$

For the block matrix using the fact that $\mathbb{1}(t = f(\boldsymbol{z}))$ is independent of β we get that

$$\frac{\partial M2}{\partial \beta_f} = \begin{pmatrix} -\frac{\partial}{\partial \beta_f^1} z^1 \pi_f(\boldsymbol{z}, \beta_{f0}) & \cdots & -\frac{\partial}{\partial \beta_f^d} z^1 \pi_f(\boldsymbol{z}, \beta_{f0}) \\ \vdots & \ddots & \vdots \\ -\frac{\partial}{\partial \beta_f^1} z^d \pi_f(\boldsymbol{z}, \beta_{f0}) & \cdots & -\frac{\partial}{\partial \beta_f^d} z^d \pi_f(\boldsymbol{z}, \beta_{f0}) \end{pmatrix}$$

$$= \{(z^i z^j)(\pi(\boldsymbol{z}, \beta_{f0}) - \pi(\boldsymbol{z}, \beta_{f0})^2)\}_{i,j}, \quad i, j \in \{1, \ldots, d\}$$

The block matrix $\frac{\partial M3}{\partial \beta_g}$ is calculated in an analogous way.

For better readability let us denote the blockmatrix in the following way:

$$\begin{pmatrix} -1 & \frac{\partial M1}{\partial \beta_f} & \frac{\partial M1}{\partial \beta_g} \\ 0 & \frac{\partial M2}{\partial \beta_f} & 0 \\ 0 & 0 & \frac{\partial M3}{\partial \beta_g} \end{pmatrix} = \begin{pmatrix} -1 & A & B \\ 0 & \partial M2 & 0 \\ 0 & 0 & \partial M3 \end{pmatrix}$$

Its inverse is given by

$$\begin{pmatrix} -1 & A \cdot \partial M2^{-1} & B \cdot \partial M3^{-1} \\ 0 & \partial M2^{-1} & 0 \\ 0 & 0 & \partial M3^{-1} \end{pmatrix}$$

and its expected value can be estimated using the sample mean. The "cheese" matrix $E\left[M(\theta_0)M(\theta_0)^T\right]$ can be estimated by the empirical mean.

3 Data generating mechanism for simulation study

3.1 Setting 1

For Setting 1 we choose:

$$P(T = 1) = expit(-Z_1)$$
$$P(T = 2) = expit(-2 + Z_1 + Z_2)$$
$$P(T = 3) = 1 - P(T = 1) - P(T = 2)$$

where the expit function is given by $expit(x) = \frac{1}{1+e^{-x}}$

Now that we have simulated $\mathbf{z}$ and t we need to simulate the outcome. The generation of the outcome Y relies on the risk function defined by:

$$P(Y = 1 \mid \boldsymbol{z}, t) = \begin{cases} 0.5 + 0.5z_1 - 0.5z_2 & \text{if } t = 1 \\ 0.65 - 0.5z_2 & \text{if } t = 2 \\ 1 - 0.5z_1 - 0.5z_2 & \text{if } t = 3 \end{cases} \tag{17}$$

The risk function is illustrated in 1. To make this data more intuitive we assume that the outcome $y = 1$ represents the unwanted outcome such as being dead and $y = 0$ the desirable outcome such as being alive.

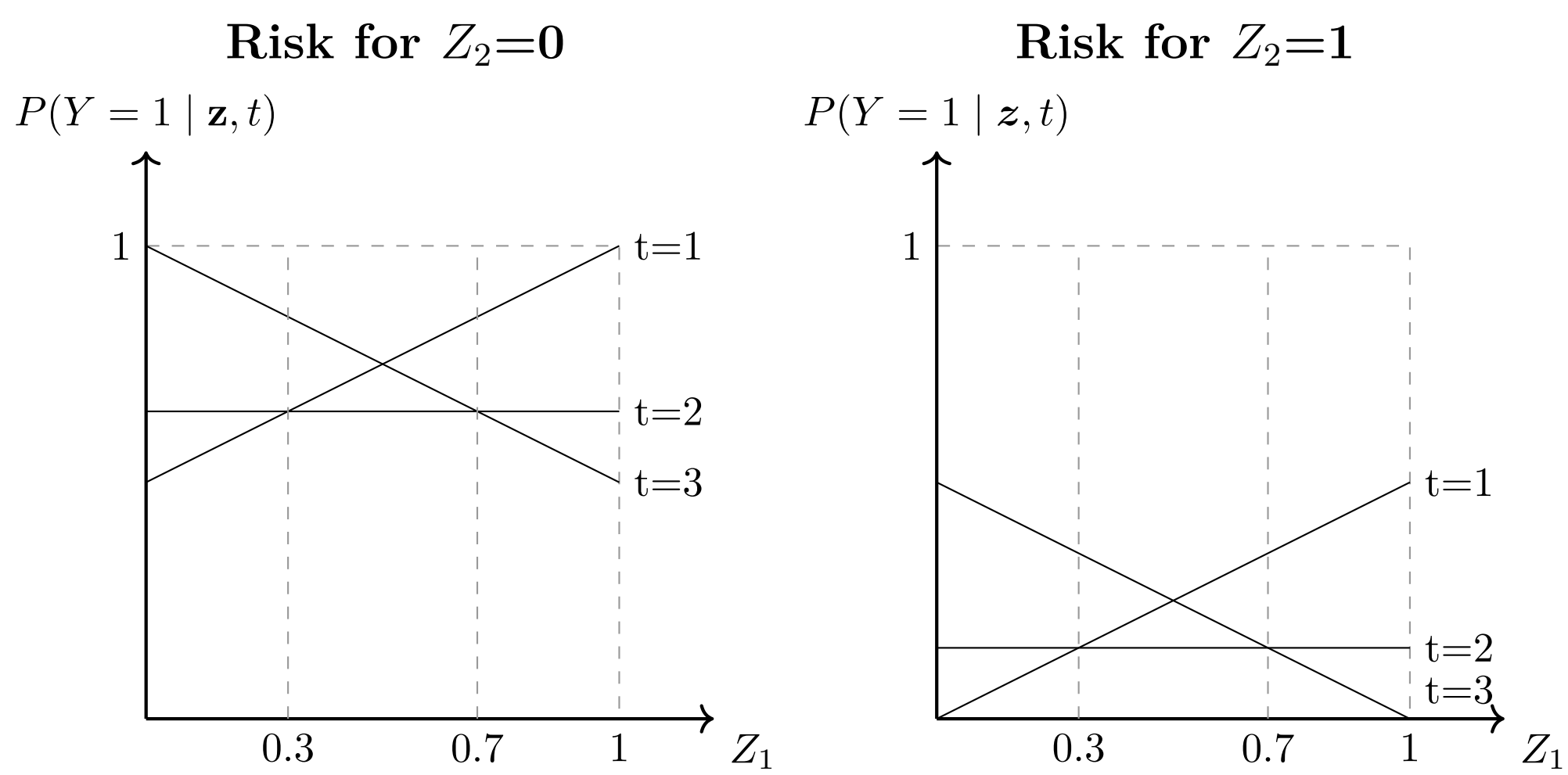

Figure 1: Risk function for the data generating mechanism for generating the probability of default $P(Y = 1 \mid \boldsymbol{z}, t)$ where each line represents one of the 3 possible treatments according to equation 17.

Last, we need a treatment regime for which we want to estimate the clinical utility. Motivated by the risk function we choose the optimal treatment regime with respect to the risk function 17. As we can read off from figure 1 the following regime has lowest probability to default. Notice this is independent of z_2:

$$f^*(\boldsymbol{z}) = f^*(z_1) := \begin{cases} 1, & \text{if } z_1 < 0.3 \\ 2, & \text{if } z_1 \in [0.3, 0.7] \\ 3, & \text{if } z_1 > 0.7 \end{cases}. \tag{18}$$

3.2 Setting 2

For Setting 2 and 3 we are using an additional parameter $s \in (-\infty, \infty)$ to control how many individuals get the optimal treatment according to the optimal treatment regime f^* in equation 18. Then probability to receive each treatment is:

$$P_s(T = t) := \begin{cases} expit(s) & \text{if } f^*(\boldsymbol{z}) = t \\ expit(g_t(\boldsymbol{z}) - s) & \text{if } f^*(\boldsymbol{z}) \neq t \end{cases},$$

where g_t is a function of $\boldsymbol{z}$ for a given t. For details we refer to the supplementary R code. We want to note that by setting s to $-\infty$ or $+\infty$ we can violate our positivity assumption. Note that this implies in the former case that no individual gets assigned the optimal treatment and in the latter case every individual is treated according to the optimal decision rule. This also implies we can set the standard of care to any value we want to investigate.

In setting 2 we choose that the treatment regime is given by

$$h(\boldsymbol{z}) = \begin{cases} 1, & \text{if } z_1 < 0.7 \\ 2, & \text{if } z_1 > 0.9 \\ 3, & \text{if } 0.7 \leq z_1 \leq 0.9 \end{cases}. \tag{19}$$

To get a nullifying result we want that our clinical utility matches that of the counterfactual outcome, hence we have run a simulation with a high number of subjects $N = 10^7$ and for $s = 1.47$ the standard of care matches the conterfactual outcome of h. Even though the standard of care and the treatment regime h assign treatments on a different basis its clinical utility is 0.

3.3 Setting 3

In setting 3 we use the same data generating mechanism as in setting 2, but this time we set $s := 1$ and choose as the treatment regime h defined in 19. The parameter $s = 1$ was chosen such that the counterfactual outcome of h lies in between the standard of care and the counterfactual outcome of the optimal treatment regime f^*. Notice that f^* is still an optimal treatment regime in Setting 3 as it is the same risk function as in Setting 1.

4 Rheumatoid arthritis example

This flowchart summarizes the steps used to construct the analytic cohort. The diagram outlines each stage of participant selection, from the initial sample to the final study population.

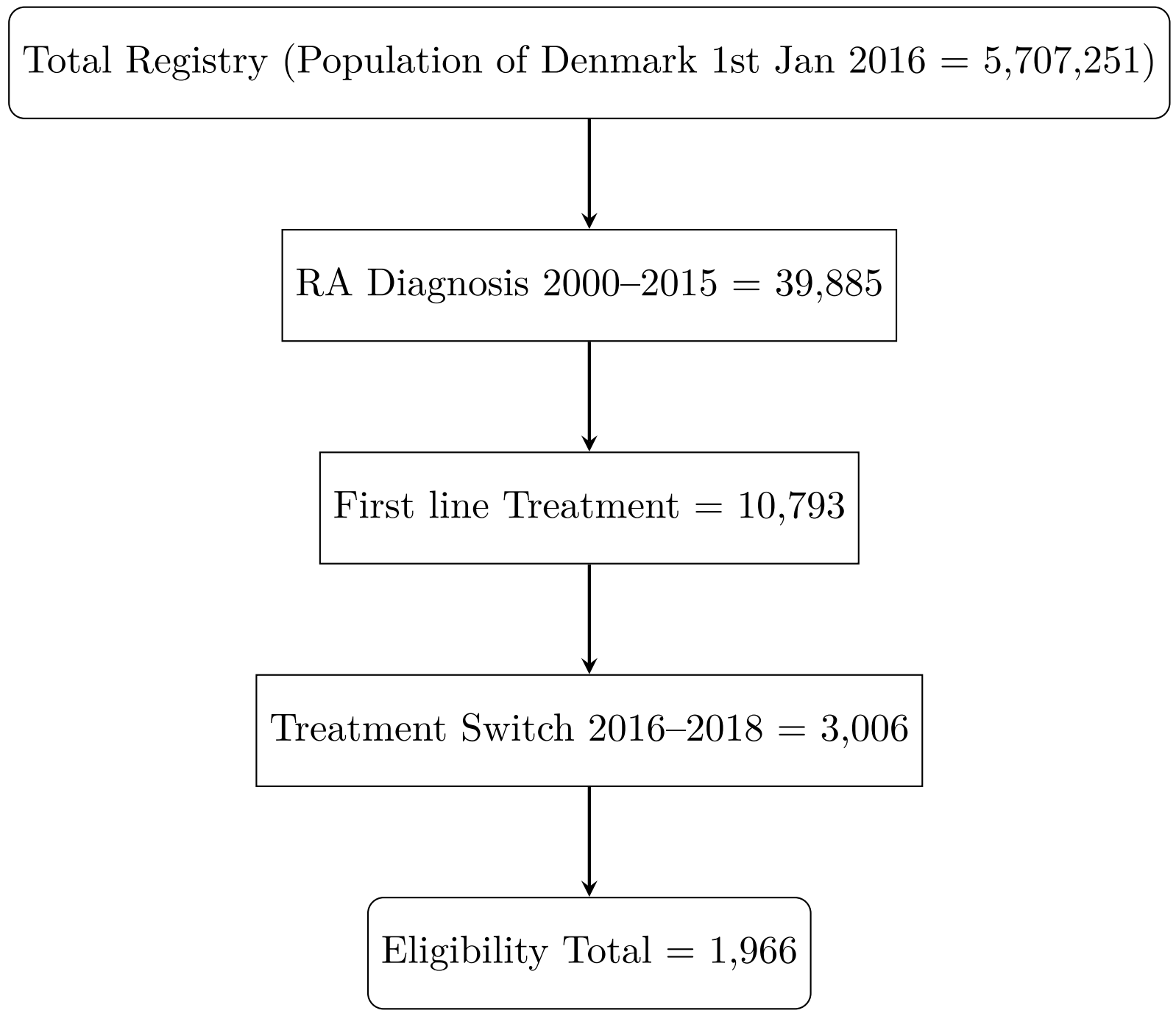

Total Registry (Population of Denmark 1st Jan 2016 = 5,707,251)
RA Diagnosis 2000–2015 = 39,885
First line Treatment = 10,793
Treatment Switch 2016–2018 = 3,006
Eligibility Total = 1,966

References

[Correa and Bareinboim(2020)] Juan Correa and Elias Bareinboim. A calculus for stochastic interventions:causal effect identification and surrogate experiments. *Proceedings of the AAAI Conference on Artificial Intelligence*, 34(06):10093–10100, Apr. 2020. doi: 10.1609/aaai.v34i06.6567. URL `https://ojs.aaai.org/index.php/AAAI/article/view/6567`.

[Stefanski and Boos(2002)] Leonard A Stefanski and Dennis D Boos. The calculus of m-estimation. *The American Statistician*, 56 (1):29–38, 2002. doi: 10.1198/000313002753631330. URL `https://doi.org/10.1198/000313002753631330`.
